# Hospital cost structure and resource utilization in unicompartmental knee arthroplasty and hip resurfacing arthroplasty: a single-center comparative cost analysis

**DOI:** 10.3389/fpubh.2026.1881345

**Published:** 2026-10-14

**Authors:** Ewa Lucka, Przemysław Daroszewski, Mateusz Lucki, Juliusz Huber

**Affiliations:** 1 Clinical Rehabilitation Laboratory, Department of Rehabilitation and Physiotherapy, University of Medical Sciences, Poznań, Poland; 2 Department of Organization and Management in Healthcare, Poznań University of Medical Sciences, Poznań, Poland; 3 Department of Pathophysiology of Locomotor Organs, Poznan University of Medical Sciences, Poznań, Poland; 4 Department and Clinic of Cardiology, University of Medical Sciences, Poznań, Poland

**Keywords:** arthroplasty, cost analysis, healthcare costs, hip resurfacing arthroplasty, unicompartmental knee arthroplasty

## Abstract

**Introduction:**

Unicompartmental knee arthroplasty (UKA) and hip resurfacing arthroplasty (HRA) are joint-preserving procedures used in different anatomical and clinical settings. Although they are not competing treatment options, both require substantial hospital resources. This study compared institutional cost structure and resource utilization associated with UKA and HRA from the hospital-provider perspective.

**Methods:**

This retrospective, single-center study included 68 unique patients undergoing UKA (*n* = 36) or HRA (*n* = 32) between January 2023 and July 2024. Each patient contributed one index hospitalization, one surgical procedure, and one independent analytical observation. The primary outcome was total index-hospitalization cost. Secondary outcomes included operating-room personnel costs, costs incurred outside the operating room, medical costs, and non-medical costs. Individual cost components and correlations with length of stay were exploratory. The association between procedure type and total index-hospitalization cost was assessed using a gamma-log generalized linear model adjusted for age, sex, body mass index, Charlson Comorbidity Index, and Kellgren–Lawrence grade 4. Secondary and exploratory comparisons were adjusted using the Benjamini–Hochberg false-discovery-rate procedure.

**Results:**

Mean total index-hospitalization cost was €5,561.53 for UKA and €6,631.18 for HRA, an unadjusted difference of €1,069.65 (*p* < 0.0001). After case-mix adjustment, HRA was associated with a 32% higher expected total index-hospitalization cost than UKA (adjusted cost ratio 1.32, 95% CI 1.07–1.63; *p* = 0.008). Medical costs were higher for HRA (€2,918.90 vs. €1,985.23; *q* < 0.0001), whereas operating-room personnel costs were higher for UKA (€371.59 vs. €335.45; *q* = 0.0035). Costs incurred outside the operating room and non-medical costs did not differ significantly. Implant costs were higher for HRA (€2,544.55 vs. €1,554.55; *q* < 0.0001). After FDR adjustment, length of stay was associated with laboratory and imaging costs in the UKA group and with laboratory costs in the HRA group.

**Conclusion:**

UKA and HRA showed distinct patterns of index-hospitalization costs and resource utilization. Higher implant expenditure was an important component of the observed cost difference. These findings represent a hospital-level comparison of two distinct arthroplasty pathways and should not be interpreted as evidence favoring either procedure. Detailed evaluation of procedure-specific cost structures may support hospital budgeting, resource allocation, implant procurement, and operational planning.

## 1 Introduction

Joint replacement procedures are increasingly used in the management of osteoarthritis, providing substantial pain relief and improvements in functional status and quality of life (1). Total hip and knee arthroplasty are well-established treatment options in clinical practice (2). However, concerns related to postoperative complications and the risk of revision surgery have contributed to continued interest in more bone-preserving approaches in appropriately selected patients (3–5). These include unicompartmental knee arthroplasty (UKA) (6–8) and hip resurfacing arthroplasty (HRA) (9, 10). UKA involves replacement of the affected compartment of the knee while preserving the remaining joint structures (11), whereas HRA replaces the articular surfaces of the hip while preserving a greater proportion of the proximal femoral bone (12, 13).

The economic burden associated with arthroplasty procedures depends on multiple factors, including implant acquisition costs, operating-room utilization, personnel requirements, duration of hospitalization, diagnostic procedures, rehabilitation, and other hospital resources. Previous economic studies have primarily evaluated UKA in comparison with total knee arthroplasty (TKA) (6–8, 14–17) and HRA in comparison with total hip arthroplasty (THA) (9, 10, 12). Other studies have compared the costs of total hip and total knee arthroplasty (18, 19). However, comparatively little attention has been paid to the detailed institutional cost structure and resource-utilization profiles of different joint-preserving arthroplasty procedures.

UKA and HRA address different joints, clinical indications, and patient populations and therefore are not competing treatment strategies. Consequently, any comparison between these procedures should be interpreted at the level of hospital resource utilization rather than as a comparison of alternative therapeutic options.

Therefore, the aim of the present study was to compare the institutional cost structure and hospital resource utilization associated with UKA and HRA during the index hospitalization. The study was not designed to determine which procedure is clinically preferable or more cost-effective, but rather to characterize differences in hospital-level resource use between two distinct joint-preserving arthroplasty pathways.

## 2 Materials and methods

The analysis included 68 unique patients undergoing UKA or HRA. Each patient contributed one index hospitalization, one surgical procedure, and one independent analytical observation. Total index-hospitalization cost comprised operating-room personnel costs, costs incurred outside the operating room, non-medical costs, and medical costs. Correlations between length of hospital stay and directly recorded medical cost components were evaluated as exploratory analyses.

### 2.1 Study design and setting

This retrospective, single-center study analyzed clinical, resource-utilization, and financial data from patients treated between January 2023 and July 2024. Ethical approval was obtained from the Bioethics Committee of Poznan University of Medical Sciences (Approval no. 176/21, issued on March 11, 2021).

The study was conducted at the Wiktor Dega Orthopedic and Rehabilitation Clinical Hospital in Poznan and included patients undergoing unicompartmental knee arthroplasty (UKA) or hip resurfacing arthroplasty (HRA). The comparison was designed as an institutional analysis of index-hospitalization costs and resource utilization. Because UKA and HRA involve different joints, clinical indications, and patient populations, they were not considered competing therapeutic alternatives. The study was therefore not designed to assess their comparative clinical effectiveness, appropriateness, or cost-effectiveness.

The final dataset comprised 68 unique patients, including 36 patients undergoing UKA and 32 undergoing HRA. Each patient had one unique medical-record identifier and contributed one index hospitalization, one surgical procedure, and one analytical observation. Thus, the analysis included 68 independent patient-level observations. No patient contributed more than one index hospitalization, surgical procedure, or analytical observation. Thus, the analysis included 68 independent patient-level observations, and no patient contributed repeated records or multiple hospitalization episodes.

Cost categories were defined according to the hospital’s standard accounting practices. An overview of the study design and analytical framework is presented in Figure 1.

**Figure 1:**
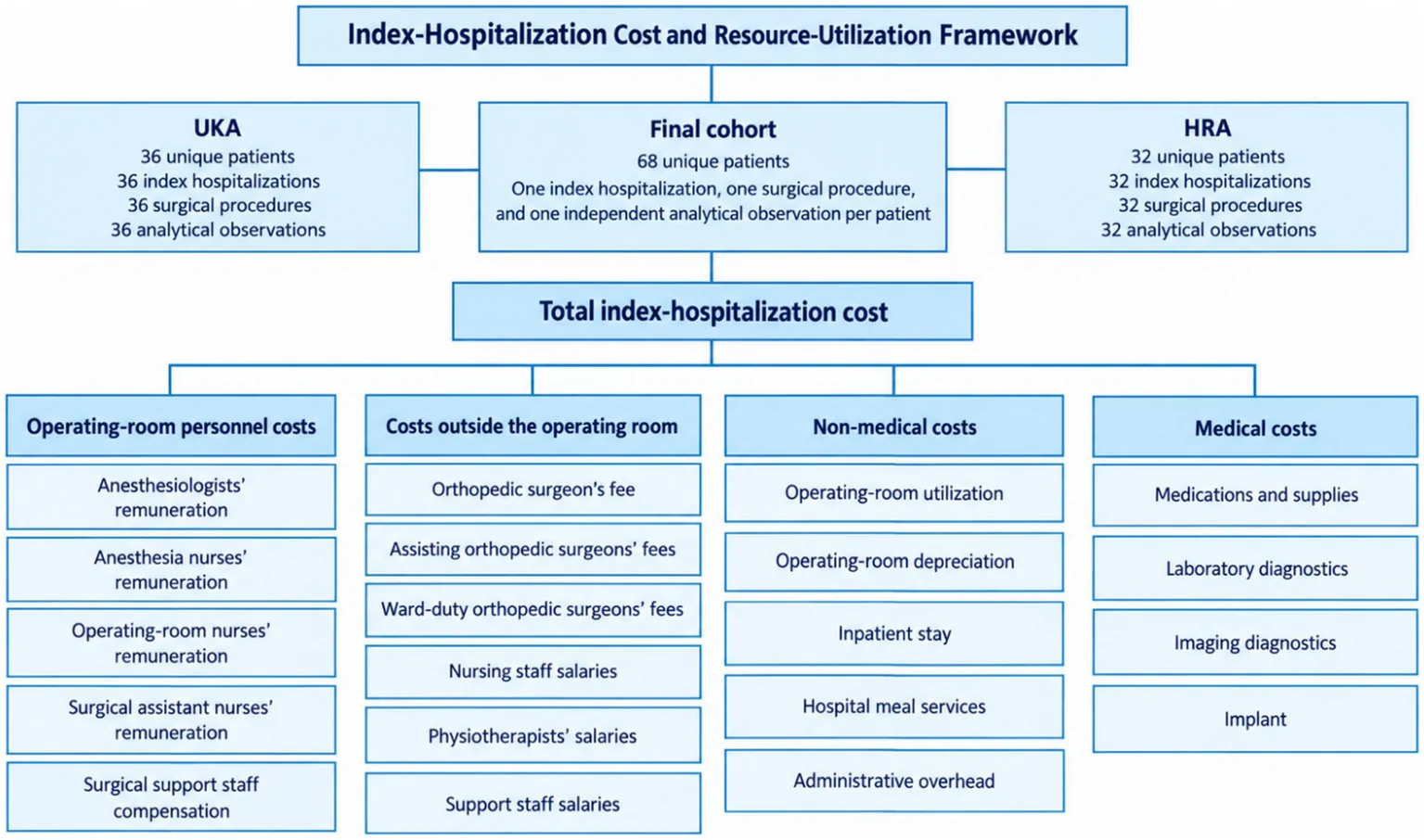
Study population and classification of index-hospitalization cost components.

### 2.2 Cost data source, costing classification, and resource utilization

The source dataset comprised patient-level hospital accounting data linked to clinical and resource-utilization data from each index hospitalization. Each patient and index hospitalization was represented by one analytical observation. The analysis was conducted from the hospital-provider perspective and included only costs incurred during the index hospitalization. The reported values represent hospital accounting costs derived from the hospital accounting system and documented patient-level resource utilization rather than patient charges or reimbursement amounts received from the payer.

The economic analysis was restricted to resource use and costs incurred during the index hospitalization. Post-discharge rehabilitation, outpatient care, subsequent admissions, revision procedures, treatment of later complications, and indirect or productivity costs were not included in the costing analysis.

Total index-hospitalization cost was calculated as the sum of operating-room personnel costs, personnel and hospitalization-related costs outside the operating room, non-medical costs, and medical costs.

Although all monetary values were obtained from the hospital accounting system, individual cost components differed according to their costing basis. Components were therefore classified as either formula-derived allocated costs or directly recorded patient-level expenditures.

Formula-derived costs were calculated within the hospital accounting system using recorded patient-level resource utilization and the applicable hospital unit rates or remuneration schedules. Operating-room personnel costs were assigned according to the applicable remuneration schedule and, where relevant, recorded operating-room utilization. Operating-room utilization and depreciation costs were allocated according to the recorded time spent in the operating suite using the corresponding hospital accounting rates. Personnel and hospitalization-related costs outside the operating room were allocated according to the applicable hospital remuneration schedules and resource-utilization measures. Physiotherapy costs were based on the documented duration of physiotherapy sessions, whereas inpatient-stay and meal-service costs were allocated according to the recorded duration of hospitalization. Administrative overhead costs represented institutional costs allocated to the individual hospitalization according to the hospital’s standard accounting methodology.

Directly recorded patient-level expenditures reflected resources or acquisition costs documented for the individual hospitalization. These components included medications and medical supplies, laboratory diagnostics, imaging diagnostics, implants, and blood products where applicable. Implant costs represented acquisition costs recorded in the hospital accounting system and were not based on reimbursement tariffs or catalog prices.

The hospital accounting data therefore included both directly attributable patient-level expenditures and allocated institutional costs. A separate economic reclassification of all cost components into fixed and variable costs was not performed. Accordingly, the present analysis should be interpreted as an analysis of full hospital accounting costs rather than a marginal-cost analysis.

This distinction was also incorporated into the interpretation of the results. Associations between a formula-derived cost and the resource measure used in its calculation were regarded as structural relationships resulting from the hospital costing method. Such associations were not interpreted as independent evidence that the resource constituted an empirically identified cost driver and were excluded from inferential correlation analyses.

Resource utilization was assessed using length of hospital stay, time spent in the operating suite, and cumulative duration of physiotherapy sessions.

Costs were analyzed in nominal Polish zloty (PLN) values as recorded in the hospital accounting system at the time of each hospitalization. No common price-year adjustment or inflation correction was applied. Accordingly, no single common price year was assigned; costs represent contemporaneous nominal accounting values recorded between January 2023 and July 2024. During the study period, from January 2023 through July 2024, no relevant changes occurred in the hospital unit rates applied to the analyzed cost components; therefore, the original accounting values were retained without additional inflation standardization.

For presentation purposes, all costs were converted from PLN to euros (€) using a single exchange rate of €1 = PLN 4.29, corresponding to the rate applied on July 25, 2024. This conversion was performed solely to express the results in a common reporting currency and was not part of the underlying costing methodology. Historical PLN-to-euro exchange-rate fluctuations were therefore not incorporated into the analysis. No discounting was applied because the analysis was limited to costs incurred during the index hospitalization.

This study was designed as a hospital-based cost and resource-utilization analysis and not as a cost-effectiveness or cost-utility analysis. Health outcomes, quality-adjusted life-years (QALYs), utilities, incremental costs, and incremental cost-effectiveness ratios were not evaluated.

### 2.3 Clinical and case-mix variables

Baseline demographic and clinical variables were used to characterize differences in case mix between the study groups. These variables included age, sex, body mass index (BMI), American Society of Anesthesiologists (ASA) physical status class, number of comorbidities, Charlson Comorbidity Index (CCI), and radiographic osteoarthritis severity according to the Kellgren–Lawrence classification. Kellgren–Lawrence grade 4 was analyzed as a binary indicator of the highest radiographic severity category.

Subsequent hospitalization during the available follow-up period of up to 2 years was recorded as a secondary clinical outcome. Because follow-up duration could differ among patients, this variable was analyzed as the occurrence of at least one subsequent hospitalization during the available observation period rather than as a standardized incidence rate.

Although the occurrence of at least one subsequent hospitalization was recorded as a descriptive secondary clinical outcome, the associated healthcare utilization and costs were not included in the economic analysis. No standardized analysis of readmission timing, cause, duration, or cost was performed.

### 2.4 Statistical analysis

Statistical analyses were performed using MedCalc software. The distributions of continuous variables were evaluated using histograms, Q–Q plots, and the Shapiro–Wilk test. Continuous variables are presented as medians with interquartile ranges (IQRs) and, where informative for financial interpretation, additionally as means with standard deviations (SDs). Categorical variables are presented as numbers and percentages.

Between-group comparisons of continuous and ordinal variables were performed using the Mann–Whitney U test. Binary categorical variables were compared using Fisher’s exact test, and the overall distribution of ASA classes was compared using the Fisher–Freeman–Halton exact test. Differences in group medians are presented with bootstrap 95% confidence intervals (CIs). For binary variables, absolute differences in proportions or odds ratios with 95% CIs are reported, as appropriate. Cramér’s V was used as the effect-size measure for the overall comparison of ASA class distributions.

Total index-hospitalization cost was defined as the primary outcome. The four aggregated cost categories—operating-room personnel costs, costs associated with hospitalization outside the operating room, medical costs, and non-medical costs—were treated as secondary outcomes. Individual cost-component comparisons and correlation analyses were considered exploratory. Baseline characteristics and resource-utilization measures were analyzed descriptively, and their reported *p* values were not included in the multiplicity correction.

To account for multiple comparisons, the Benjamini–Hochberg false discovery rate (FDR) procedure was applied separately within three defined families of secondary and exploratory analyses: the four aggregated cost categories presented in Table 1, the 17 individual cost-component comparisons presented in Table 2, and the six correlation analyses presented in Table 3. Variables with identical values and no variability in either group were not statistically compared and were excluded from the FDR procedure. Both nominal *p* values and FDR-adjusted q values are reported. An FDR-adjusted q value <0.05 was considered statistically significant. Findings from exploratory analyses were interpreted as hypothesis-generating.

**Table 1:** Distribution of index-hospitalization costs by procedure type.

Cost category	UKA index hospitalizations		HRA index hospitalizations		*p* value	FDR-adjusted q value
	mean ± SD, €	median [Q1–Q3], €	mean ± SD, €	median [Q1–Q3], €		
Operating-room personnel costs	371.59 ± 49.55	378.13 [337.83–401.79]	335.45 ± 33.58	337.83 [317.04–353.18]	0.0017	0.0035
Costs associated with hospitalization outside the operating room	1901.83 ± 433.28	1796.42 [1648.93–2087.96]	2043.82 ± 798.39	1596.74 [1561.29–2246.27]	0.117	0.156
Non-medical costs	1302.88 ± 269.69	1268.62 [1086.30–1488.93]	1333.01 ± 455.89	1079.50 [1024.50–1621.20]	0.353	0.353
Medical costs	1985.23 ± 576.85	2033.61 [1344.47–2194.75]	2918.90 ± 129.00	2929.29 [2825.45–3027.48]	<0.0001	<0.0001
Total index hospitalization costs — primary outcome	5561.53 ± 803.82	5282.73 [5037.03–6028.61]	6631.18 ± 1273.93	5974.10 [5882.60–7104.55]	<0.0001	Not applicable

Each index hospitalization corresponded to one unique patient and one surgical procedure. Data are presented as mean ± standard deviation (SD) and median [Q1–Q3]. Between-group comparisons were performed using the Mann–Whitney U test. Total index-hospitalization cost was the prespecified primary outcome and was evaluated using an unadjusted *p* value; therefore, an FDR-adjusted q value was not applicable. The four aggregated cost categories were considered secondary outcomes, and their nominal *p* values were adjusted jointly for multiple comparisons using the Benjamini–Hochberg false discovery rate procedure. An FDR-adjusted q value <0.05 was considered statistically significant. HRA, hip resurfacing arthroplasty; UKA, unicompartmental knee arthroplasty; SD, standard deviation; Q1, first quartile; Q3, third quartile; FDR, false discovery rate.

**Table 2:** Exploratory comparison of individual index-hospitalization cost components between the UKA and HRA groups.

Cost component	UKA		HRA		*p* value	FDR-adjusted *q* value
	Mean ± SD, €	median [Q1–Q3], €	Mean ± SD, €	Median [Q1–Q3], €		
Operating-room personnel costs						
Anesthesiologists’ remuneration ^	137.05 ± 30.10	141.03 [116.55–155.40]	115.19 ± 20.26	116.55 [103.92–125.87]	0.0019	0.0066
Anesthesia nurse’s remuneration ^	52.50 ± 0.00	52.50 [52.50–52.50]	52.50 ± 0.00	52.50 [52.50–52.50]	Not calculated	Not applicable
Operating room nurse’s remuneration^	46.72 ± 0.00	46.72 [46.72–46.72]	46.72 ± 0.00	46.72 [46.72–46.72]	Not calculated	Not applicable
Surgical assistant nurse’s remuneration^	46.72 ± 0.00	46.72 [46.72–46.72]	46.72 ± 0.00	46.72 [46.72–46.72]	Not calculated	Not applicable
Compensation for surgical support staff ^	88.60 ± 19.46	91.17 [75.34–100.46]	74.33 ± 13.31	75.34 [67.18–81.37]	0.0017	0.0066
Costs associated with hospitalization outside the operating room						
Orthopedic surgeon’s fee^	450.38 ± 0.02	450.38 [450.37–450.38]	385.27 ± 13.71	382.43 [371.97–400.09]	<0.0001	<0.0001
Orthopedic surgeons’ fees for assisting^	57.43 ± 29.03	67.58 [52.54–76.15]	56.44 ± 9.93	57.11 [50.92–61.68]	0.069	0.130
Orthopedic surgeons’ fees for hospital ward duties outside the operating room^	446.68 ± 158.02	386.04 [356.07–498.50]	498.67 ± 255.76	356.07 [356.07–535.12]	0.424	0.515
Nursing staff salaries^	646.40 ± 180.14	614.29 [542.23–759.12]	770.98 ± 387.44	542.23 [542.23–884.50]	0.767	0.767
Salaries of physiotherapists^	147.88 ± 42.87	156.58 [111.84–156.58]	160.43 ± 79.91	111.84 [111.84–190.85]	0.613	0.695
Salaries of support staff^	153.06 ± 55.09	132.95 [118.74–166.24]	172.04 ± 85.06	118.74 [118.74–212.91]	0.317	0.414
Non-medical costs						
Operating room utilization costs^	375.39 ± 82.43	386.27 [319.23–425.64]	315.19 ± 55.99	319.23 [284.65–344.77]	0.0019	0.0066
Operating room depreciation costs^	24.67 ± 5.42	25.38 [20.98–27.97]	21.38 ± 3.03	20.98 [20.28–22.66]	0.0037	0.0090
Cost of stay during hospitalization^	624.26 ± 182.42	656.35 [468.82–762.45]	705.13 ± 348.13	468.82 [468.82–995.89]	0.767	0.767
Cost of hospital meal services^	71.34 ± 22.45	73.75 [54.35–76.09]	75.99 ± 39.07	54.35 [54.35–80.95]	0.229	0.340
Administrative overhead costs^	207.22 ± 67.55	199.12 [151.94–207.31]	215.33 ± 106.26	148.08 [148.08–270.27]	0.133	0.225
Medical costs						
Cost of medications and supplies^^	301.06 ± 75.93	291.16 [241.23–357.02]	246.82 ± 42.43	252.49 [223.97–281.71]	0.0023	0.0066
Cost of laboratory diagnostics^^	106.36 ± 22.02	108.31 [85.32–116.36]	111.54 ± 18.81	114.39 [97.16–122.71]	0.240	0.340
Cost of imaging diagnostics ^^	23.26 ± 12.43	23.26 [11.63–34.90]	15.99 ± 8.22	11.63 [11.63–14.54]	0.0079	0.0169
Cost of the implant^^	1554.55 ± 572.82	1560.84 [981.82–1711.89]	2544.55 ± 100.43	2546.43 [2447.62–2643.36]	<0.0001	<0.0001

Each observation represented one unique patient, one index hospitalization, and one surgical procedure. Data are presented as mean ± standard deviation (SD) and median [Q1–Q3]. Between-group comparisons were performed using the Mann–Whitney U test. These analyses were considered exploratory. Nominal *p* values were adjusted jointly across 17 comparisons using the Benjamini–Hochberg false discovery rate (FDR) procedure. Statistical comparisons were not performed for variables with identical values and no variability in either group. Cost components were classified according to their costing basis. ^ Formula-derived allocated costs were calculated within the hospital accounting system using documented patient-level resource utilization and the applicable hospital unit rate or remuneration schedule. ^^ Directly recorded patient-level expenditures reflected documented resource use or acquisition costs attributable to the individual index hospitalization. FDR-adjusted q values were not applicable to these variables. HRA, hip resurfacing arthroplasty; UKA, unicompartmental knee arthroplasty; SD, standard deviation; Q1, first quartile; Q3, third quartile; FDR, false discovery rate.

**Table 3:** Exploratory correlations between length of hospital stay and selected medical cost components.

Study group	Medical cost component	Spearman’s r	*p* value	FDR-adjusted *q* value
UKA	Medications and supplies	−0.296	0.079	0.095
	Laboratory diagnostics	−0.402	0.015	0.030
	Imaging diagnostics	0.530	0.001	0.003
HRA	Medications and supplies	0.330	0.065	0.095
	Laboratory diagnostics	0.824	<0.0001	<0.0006
	Imaging diagnostics	−0.266	0.142	0.142

Correlations were calculated at the patient level. Each patient contributed one index hospitalization and one analytical observation. Spearman rank correlation coefficients are presented. The analyses were considered exploratory. Nominal *p* values were adjusted jointly across six correlations using the Benjamini–Hochberg false discovery rate (FDR) procedure. An FDR-adjusted q value <0.05 was considered statistically significant. Only cost components based on the actual use of medications, supplies, and diagnostic procedures were included. Costs calculated directly as length of stay multiplied by a predetermined daily rate were excluded. HRA, hip resurfacing arthroplasty; UKA, unicompartmental knee arthroplasty; FDR, false discovery rate.

Spearman’s rank correlation coefficient was used to examine associations between length of hospital stay and selected directly recorded medical cost components within each procedure group. The six exploratory correlations included medications and medical materials, laboratory diagnostics, and imaging diagnostics, analyzed separately in the UKA and HRA groups. Their nominal *p* values were adjusted jointly using the Benjamini–Hochberg false discovery rate procedure.

Formula-derived cost components were excluded from inferential correlation analyses when the analyzed resource measure was used directly in their calculation. In particular, correlations between operating-room duration and time-based personnel or operating-room costs, as well as correlations between length of hospital stay and costs allocated using daily unit rates, were considered mathematically determined by the accounting formulas. These relationships were therefore not subjected to hypothesis testing and were not interpreted as independently identified cost drivers.

Because total index-hospitalization costs were strictly positive and positively skewed, the adjusted association between procedure type and total index-hospitalization cost was evaluated using a generalized linear model with a gamma distribution and log link. Procedure type was the principal explanatory variable, and the model was adjusted for age, sex, BMI, Charlson Comorbidity Index, and Kellgren–Lawrence grade 4. UKA was the reference category. Because each patient contributed only one analytical observation, observations were treated as independent. Model coefficients were exponentiated and reported as adjusted cost ratios with 95% confidence intervals. A cost ratio greater than 1 indicated a higher expected total index-hospitalization cost for HRA relative to UKA, conditional on the covariates included in the model.

A stratified nonparametric bootstrap with 5,000 replications was performed as a sensitivity analysis of the adjusted procedure effect. Individual patient observations were resampled with replacement separately within the UKA and HRA groups. The bootstrap estimate is reported as a cost ratio with a percentile 95% confidence interval.

All statistical tests were two-sided. For the primary adjusted analysis, *p* < 0.05 was considered statistically significant. Secondary and exploratory findings were interpreted using their corresponding FDR-adjusted *q* values.

## 3 Results

The final analytical cohort included 68 unique patients: 36 undergoing UKA and 32 undergoing HRA. Each patient had one unique medical-record identifier and contributed one index hospitalization, one surgical procedure, and one independent analytical observation. No patient contributed more than one index hospitalization, surgical procedure, or analytical observation.

The characteristics of the study groups are presented in Table 4. Patients undergoing HRA were younger than those undergoing UKA [median 50.5 (IQR 46.3–54.8) vs. 58.5 (IQR 54.0–61.0) years; median difference −8.0 years, 95% CI − 11.5 to −4.0; *p* = 0.001] and had a lower BMI [27.44 (IQR 26.42–29.40) vs. 31.34 (IQR 29.74–34.87) kg/m^2^; median difference −3.90 kg/m^2^, 95% CI − 5.78 to −2.02; *p* < 0.001]. All patients in the HRA group were male, compared with 72.2% of patients in the UKA group (absolute difference, 27.8 percentage points; 95% CI, 11.7–44.0; Fisher’s exact *p* = 0.001).

**Table 4:** Characteristics of the study groups.

Characteristic	UKA patients (*n* = 36)	HRA patients (*n* = 32)	Effect estimate, HRA vs. UKA (95% CI)	*p* value
Demographic and clinical characteristics				
Male sex, *n* (%)	26 (72.2)	32 (100.0)	Difference 27.8 pp. (11.7 to 44.0)	0.001
Age, years, median [IQR]	58.5 [54.0–61.0]	50.5 [46.3–54.8]	Median difference −8.0 years (−11.5 to −4.0)	0.001
BMI, kg/m^2^, median [IQR]	31.34 [29.74–34.87]	27.44 [26.42–29.40]	Median difference −3.90 kg/m^2^ (−5.78 to −2.02)	<0.001
ASA class, *n* (%)				
ASA I	8 (22.2)	8 (25.0)	Cramér’s V = 0.27	0.065
ASA II	28 (77.8)	20 (62.5)		
ASA III	0 (0.0)	4 (12.5)		
Number of comorbidities, median [IQR]	1.0 [0–3.0]	0.5 [0–1.5]	Median difference −0.5 (−2.0 to 1.0)	0.178
Charlson comorbidity index, median [IQR]	0 [0–2.0]	0 [0–0.5]	Median difference 0 (−1.5 to 0)	0.076
Kellgren–Lawrence grade 4, *n* (%)	12 (33.3)	4 (12.5)	OR 0.29 (0.08–1.00)	0.051
Perioperative resource utilization				
Length of hospital stay, days, median [IQR]	5 [5–8.25]	7 [5–7]	Median difference 2.0 days (0 to 2.0)	0.385
Time spent in the operating suite, min, median [IQR]	181.5 [150–200]	150 [133.75–162]	Median difference −31.5 min (−47.5 to −10.0)	0.002
Duration of physiotherapy sessions, h, median [IQR]	3.5 [2.5–3.5]	2.5 [2.5–4.63]	Median difference −1.0 h (−1.0 to 0.67)	0.613
Follow-up outcome				
Any subsequent hospitalization during available follow-up, *n* (%)	16 (44.4)	8 (25.0)	OR 0.42 (0.15–1.17)	0.129

Data are presented at the patient level. Each patient contributed one index hospitalization and one surgical procedure. Data are presented as *n* (%) or median [interquartile range], as indicated. Continuous and ordinal variables were compared using the Mann–Whitney U test because their distributions departed from normality in at least one study group. Binary categorical variables were compared using Fisher’s exact test, and the overall ASA distribution was compared using the Fisher–Freeman–Halton exact test. Effect estimates for continuous variables are presented as differences in group medians with bootstrap 95% confidence intervals. For binary variables, odds ratios or absolute differences in proportions with 95% confidence intervals are reported, as appropriate. Effect estimates are expressed for HRA relative to UKA. Radiographic osteoarthritis severity was classified according to Kellgren–Lawrence; grades 1–3 were grouped together and grade 4 was considered advanced radiographic osteoarthritis. Abbreviations: ASA, American Society of Anesthesiologists; BMI, body mass index; HRA, hip resurfacing arthroplasty; IQR, interquartile range; SD, standard deviation; UKA, unicompartmental knee arthroplasty.

The overall distribution of ASA classes did not differ significantly between the groups (Fisher–Freeman–Halton *p* = 0.065; Cramér’s V = 0.27). No statistically significant differences were observed in the number of comorbidities, Charlson Comorbidity Index, prevalence of Kellgren–Lawrence grade 4 osteoarthritis, or subsequent hospitalization during the available follow-up period.

The median time spent in the operating suite was longer in the UKA group than in the HRA group [181.5 (IQR 150–200) vs. 150 (IQR 133.75–162) minutes; median difference for HRA versus UKA − 31.5 min, 95% CI − 47.5 to −10.0; *p* = 0.002]. In contrast, neither length of hospital stay (*p* = 0.385) nor cumulative duration of physiotherapy sessions (*p* = 0.613) differed significantly between the groups.

The distribution of index-hospitalization costs by procedure type is presented in Table 1. The primary outcome, total index-hospitalization cost, was significantly higher in the HRA group than in the UKA group [mean: €6,631.18 vs. €5,561.53; median (Q1–Q3): €5,974.10 (€5,882.60–€7,104.55) vs. €5,282.73 (€5,037.03–€6,028.61); unadjusted *p* < 0.0001]. The unadjusted difference in mean total index-hospitalization cost was €1,069.65.

Among the four secondary cost categories, medical costs were significantly higher in the HRA group than in the UKA group (mean: €2,918.90 vs. €1,985.23; *p* < 0.0001; FDR-adjusted *q* < 0.0001), corresponding to a mean difference of €933.67. Conversely, operating-room personnel costs were significantly higher in the UKA group than in the HRA group (mean: €371.59 vs. €335.45; *p* = 0.0017; FDR-adjusted *q* = 0.0035), corresponding to a mean difference of €36.14. No statistically significant between-group differences were observed in costs associated with hospitalization outside the operating room (*p* = 0.117; *q* = 0.156) or in non-medical costs (*p* = 0.353; *q* = 0.353). Thus, the differences in medical costs and operating-room personnel costs remained statistically significant after correction for multiple comparisons.

Given the differences in baseline characteristics between the UKA and HRA groups, a multivariable generalized linear model with a gamma distribution and log link was used to evaluate the association between procedure type and total index-hospitalization cost (Table 5). After adjustment for age, sex, BMI, Charlson Comorbidity Index, and Kellgren–Lawrence grade 4, HRA was associated with a significantly higher expected total index-hospitalization cost compared with UKA (adjusted cost ratio 1.32, 95% CI 1.07–1.63; *p* = 0.008). This estimate corresponds to an approximately 32% higher expected cost for HRA.

**Table 5:** Multivariable generalized linear model of total index hospitalization cost.

Predictor	Adjusted cost ratio	95% CI	*p* value
Procedure: HRA vs. UKA	1.32	1.07–1.63	0.008
Age, per 1-year increase	1.008	1.000–1.015	0.056
Male sex	1.02	0.84–1.23	0.86
BMI, per 1 kg/m^2^ increase	1.015	0.990–1.041	0.23
Charlson comorbidity index, per 1-point increase	0.992	0.959–1.027	0.64
Kellgren–Lawrence grade 4	1.04	0.90–1.20	0.61

Adjusted cost ratios were estimated using a generalized linear model with a gamma distribution and log link based on 68 independent patient-level observations. The model included procedure type, age, sex, BMI, Charlson Comorbidity Index, and Kellgren–Lawrence grade 4. UKA was the reference category. Each patient contributed one index hospitalization, one surgical procedure, and one analytical observation. A stratified patient-level nonparametric bootstrap with 5,000 replications was performed as a sensitivity analysis. Abbreviations: BMI, body mass index; CI, confidence interval; HRA, hip resurfacing arthroplasty; UKA, unicompartmental knee arthroplasty.

Age was not significantly associated with total index-hospitalization cost (adjusted cost ratio per 1-year increase, 1.008; 95% CI, 1.000–1.015; *p* = 0.056). Sex, BMI, Charlson Comorbidity Index, and Kellgren–Lawrence grade 4 were also not independently associated with total index-hospitalization cost (all *p* > 0.05).

A stratified patient-level nonparametric bootstrap with 5,000 replications yielded a similar estimate for HRA versus UKA (cost ratio 1.29, 95% percentile CI 1.15–1.55; *p* = 0.0015). The direction and magnitude of the bootstrap estimate were consistent with those obtained in the primary model. Variance inflation factors ranged from 1.38 to 2.69, indicating no evidence of severe multicollinearity.

An exploratory analysis of individual index-hospitalization cost components is presented in Table 2. After adjustment for multiple comparisons using the Benjamini–Hochberg false discovery rate procedure, two differences in operating-room personnel costs remained statistically significant. Mean anesthesiologists’ remuneration was €21.86 higher in the UKA group than in the HRA group (€137.05 vs. €115.19; *p* = 0.0019; *q* = 0.0066), while mean compensation for surgical support staff was €14.27 higher (€88.60 vs. €74.33; *p* = 0.0017; *q* = 0.0066). Statistical comparisons were not performed for the three nursing cost components because these variables had identical values and no variability in either group.

Among personnel costs associated with hospitalization outside the operating room, the orthopedic surgeon’s fee was €65.11 higher in the UKA group than in the HRA group (€450.38 vs. €385.27; *p* < 0.0001; *q* < 0.0001). No statistically significant differences were observed for surgeons’ assisting fees, physicians’ ward duties, nursing staff salaries, physiotherapists’ salaries, or support staff salaries after FDR adjustment.

Among non-medical costs, operating-room utilization costs were €60.20 higher in the UKA group (€375.39 vs. €315.19; *p* = 0.0019; *q* = 0.0066), while operating-room depreciation costs were €3.29 higher (€24.67 vs. €21.38; *p* = 0.0037; *q* = 0.0090). No statistically significant differences were observed in hospitalization, meal-service, or administrative overhead costs.

Regarding medical costs, the UKA group had higher costs of medications and supplies by €54.24 (€301.06 vs. €246.82; *p* = 0.0023; *q* = 0.0066) and higher imaging diagnostic costs by €7.27 (€23.26 vs. €15.99; *p* = 0.0079; *q* = 0.0169). Conversely, implant costs were €990.00 higher in the HRA group than in the UKA group (€2,544.55 vs. €1,554.55; *p* < 0.0001; *q* < 0.0001). Laboratory diagnostic costs did not differ significantly between the groups. Given the exploratory nature of these component-level analyses, the findings should be interpreted cautiously.

Exploratory correlations between length of hospital stay and selected medical cost components are presented in Table 3. After FDR adjustment, length of stay was negatively correlated with laboratory diagnostic costs in the UKA group (Spearman’s *r* = −0.402, *q* = 0.030) and positively correlated with imaging diagnostic costs (Spearman’s *r* = 0.530, *q* = 0.003). In the HRA group, length of stay was strongly positively correlated with laboratory diagnostic costs (Spearman’s *r* = 0.824, *q* < 0.0006). The remaining correlations did not remain statistically significant after FDR adjustment. These findings should be interpreted as exploratory and hypothesis-generating.

## 4 Discussion

In recent years, bone-preserving arthroplasty procedures, including unicompartmental knee arthroplasty (UKA) and hip resurfacing arthroplasty (HRA), have remained relevant treatment options in appropriately selected patients (20, 21). In the present study, patients undergoing HRA were younger, had a lower BMI, and were exclusively male, whereas the UKA group was older and included both women and men (Table 4). These differences most likely reflect the distinct clinical indications, eligibility criteria, and patient-selection patterns associated with each procedure rather than a direct preference for one intervention over the other.

The rationale for this cross-joint comparison was administrative and economic rather than clinical. UKA and HRA are not competing treatment options for the same condition; therefore, a direct comparison of their clinical effectiveness would be inappropriate. From the hospital-provider perspective, however, both procedures use overlapping categories of institutional resources, including operating-room infrastructure, surgical and anesthesiology personnel, implants, diagnostics, inpatient care, and rehabilitation services. Comparing their resource and cost profiles may therefore support hospital budgeting, implant procurement, staffing, operating-room scheduling, and capacity planning. Accordingly, the findings should be interpreted within an institutional cost-structure and resource-utilization framework and not as evidence favoring either procedure for clinical use.

The mean total index-hospitalization cost was lower for UKA than for HRA (€5,561.53 vs. €6,631.18, respectively; Table 1). Because the procedures involve different joints, indications, patient populations, and resource requirements, this difference should not be interpreted as evidence of greater clinical or economic value of UKA. Rather, it demonstrates that two distinct arthroplasty pathways may generate different levels and patterns of hospital expenditure.

The adjusted gamma-log generalized linear model further indicated that the difference in total hospitalization cost was not explained solely by the measured differences in baseline case mix. After adjustment, HRA was associated with an approximately 32% higher expected total index-hospitalization cost than UKA. This estimate represents a conditional difference in hospital expenditure after accounting for the covariates included in the model and should not be interpreted causally. Residual confounding related to unmeasured clinical, procedural, and organizational factors remains possible.

Previous studies have similarly shown that cost structures may differ substantially among arthroplasty procedures. Palsis et al. (19), in an analysis of total hip and total knee arthroplasty, identified differences in both overall costs and individual cost components between hip and knee replacement procedures. Likewise, Liao et al. (18) reported differences in healthcare utilization and costs between total hip and total knee arthroplasty. Although these studies examined different procedures and healthcare settings, they support the broader observation that procedure-specific requirements may substantially influence hospital expenditure.

Economic evaluations conducted within the same anatomical and clinical treatment pathway have additionally demonstrated the importance of considering both costs and patient outcomes. Clarke et al. (12) and Edlin et al. (9) evaluated hip resurfacing compared with total hip arthroplasty, whereas Beard et al. (8) assessed unicompartmental versus total knee arthroplasty. Edlin et al. (9) reported differences in costs and quality-adjusted life-years between hip resurfacing and total hip arthroplasty, while Beard et al. (8) demonstrated favorable longer-term economic outcomes for UKA compared with total knee arthroplasty. However, these studies compared alternative interventions for the same joint and clinical condition. They therefore differ fundamentally from the present analysis, which was designed to compare hospital costs and resource use across two distinct arthroplasty pathways and did not assess comparative cost-effectiveness. Therefore, the observed differences reflect index-hospitalization expenditure only and cannot be extrapolated to the overall economic burden of the complete episode of care or to longer-term cost-effectiveness.

In the present cohort, the median length of hospital stay was 5 days [IQR 5–8.25] after UKA and 7 days [IQR 5–7] after HRA; however, the between-group difference was not statistically significant (*p* = 0.385). Therefore, length of stay should be interpreted descriptively and not as evidence of greater clinical efficiency of either procedure. Previous studies comparing UKA with total knee arthroplasty have identified length of stay as an important component of resource utilization. Maman et al. (7), for example, reported shorter hospital stays and lower costs for UKA than for total knee arthroplasty. Because UKA and HRA involve different joints and differently selected patient populations, however, these findings cannot be directly extrapolated to the present cross-joint comparison.

The analysis of individual cost components (Table 2) provided further insight into the mechanisms underlying the difference in total expenditure. Implant-related expenditure was higher for HRA and represented an important contributor to its higher total index-hospitalization cost. Conversely, several operating-room and other hospital cost components were higher for UKA, demonstrating that the direction of cost differences was not uniform across all categories. Thus, the difference in total index-hospitalization cost should not be attributed to a single resource component.

Previous studies have likewise emphasized the contribution of implant prices and other procedure-specific resources to the overall cost of arthroplasty. Chisari et al. (22), comparing unicompartmental and total knee arthroplasty, reported differences in both implant and overall facility costs. Palsis et al. (19) also demonstrated variation in personnel, space and equipment, and indirect costs between total hip and total knee arthroplasty. Collectively, these findings show that total hospital expenditure reflects the combined effects of implant costs, operating-room use, staffing, diagnostics, inpatient care, and other institutional resources.

The present findings underline the value of detailed institutional cost analysis. Rather than informing the clinical selection of UKA versus HRA, the decomposition of total expenditure into individual cost components may help identify procedure-specific opportunities for more efficient resource allocation, implant procurement, operating-room planning, staffing, and cost control. Such information may be particularly relevant to hospitals performing multiple arthroplasty procedures with distinct clinical and resource profiles. Nevertheless, any cost-management strategy should preserve appropriate patient selection, procedural quality, and clinical outcomes.

## 5 Limitations of the study

Several limitations should be acknowledged. First, this was a retrospective, single-center study with a relatively small sample of 68 unique patients, including 36 patients undergoing UKA and 32 undergoing HRA. The retrospective design may have introduced selection bias and limited the availability of potentially relevant clinical, procedural, and organizational variables. The relatively small sample also limits the precision of effect estimates and the number of covariates that can be reliably included in multivariable analyses.

Second, the UKA and HRA groups differed substantially in baseline characteristics, including age, sex, and BMI, reflecting the distinct clinical indications and patient-selection patterns for these procedures. Although the adjusted analysis accounted for age, sex, BMI, Charlson Comorbidity Index, and Kellgren–Lawrence grade 4, residual confounding from unmeasured or incompletely measured case-mix factors cannot be excluded. Consequently, the observed differences in index-hospitalization costs should not be interpreted as being caused solely by the type of surgical procedure.

Third, the economic analysis was restricted to costs incurred during the index hospitalization and therefore does not represent the total cost of care over the complete treatment pathway. Post-discharge rehabilitation, outpatient care, subsequent hospitalizations, revision procedures, treatment of complications occurring after discharge, and other longer-term healthcare utilization were not included in the costing analysis. Although the occurrence of at least one subsequent hospitalization was recorded descriptively, the timing, indication, duration, and costs of these hospitalizations were not evaluated.

Fourth, the study did not assess clinical effectiveness or patient-centered outcomes. Data on postoperative complications, functional outcomes, health-related quality of life, utilities, quality-adjusted life-years (QALYs), or incremental cost-effectiveness ratios were not available. Accordingly, this study represents a hospital-provider cost and resource-utilization analysis rather than a cost-effectiveness or cost-utility analysis, and the observed cost differences should not be interpreted as evidence of superior clinical or economic value of either procedure.

Fifth, several cost components were formula-derived within the hospital accounting system using recorded resource utilization and predetermined unit rates. Therefore, associations between these cost components and the corresponding resource measures may partly reflect the underlying accounting formulas rather than independent empirical relationships. For this reason, formula-derived cost components were excluded from inferential correlation analyses when the analyzed resource measure directly entered their calculation.

Sixth, indirect costs, including productivity losses, informal care, and other societal costs, were outside the hospital-provider perspective and were not assessed.

Seventh, the study included multiple secondary and exploratory comparisons, increasing the risk of Type I error. Although the Benjamini–Hochberg false discovery rate procedure was applied within predefined families of analyses, the component-level findings should be interpreted as exploratory and hypothesis-generating.

Finally, because the costing structure, remuneration schedules, accounting methods, implant procurement practices, and healthcare financing arrangements are institution- and system-specific, the absolute cost estimates and some observed cost patterns may not be directly generalizable to other hospitals or healthcare systems.

## 6 Conclusion

UKA and HRA were associated with distinct patterns of index-hospitalization costs and resource utilization at our institution. The mean total index-hospitalization cost was higher for HRA, and this difference persisted after adjustment for measured baseline characteristics. Higher implant expenditure was an important component of the observed cost difference, although other cost components showed procedure-specific patterns. These findings represent an institutional comparison of two distinct arthroplasty pathways and should not be interpreted as evidence of the clinical or economic superiority of either procedure. Nevertheless, detailed analysis of procedure-specific cost structures may support hospital budgeting, resource allocation, implant procurement, and operational planning. These findings are limited to the index hospitalization and do not provide evidence regarding longer-term healthcare costs, clinical effectiveness, or cost-effectiveness.

## Data Availability

The raw data supporting the conclusions of this article will be made available by the authors, without undue reservation.
